# Research Progress on Flavonoids in Traditional Chinese Medicine to Counteract Cardiotoxicity Associated with Anti-Tumor Drugs

**DOI:** 10.31083/j.rcm2503074

**Published:** 2024-02-27

**Authors:** Hongwei Shi, Lian Duan, Li Tong, Peng Pu, Lai Wei, Linlin Wang, Desheng Hu, Heng Tang

**Affiliations:** ^1^Department of Radiation Oncology, Hubei Cancer Hospital, Tongji Medical College, Huazhong University of Science and Technology, 430030 Wuhan, Hubei, China; ^2^Department of Oncology, Renmin Hospital of Wuhan University, 430064 Wuhan, Hubei, China; ^3^Department of Cardiology, The First Affiliated Hospital of Chongqing Medical University, 400016 Chongqing, China; ^4^Department of Pharmacy, Hubei Cancer Hospital, Tongji Medical College, Huazhong University of Science and Technology, 430030 Wuhan, Hubei, China; ^5^Department of Radiation Oncology, Shandong Cancer Hospital and Institute, Shandong First Medical University and Shandong Academy of Medical Sciences, 250117 Jinan, Shandong, China; ^6^Department of Cardiology, Southwest Hospital, Third Military Medical University (Army Medical University), 400038 Chongqing, China

**Keywords:** traditional Chinese medicine, Flavonoid, anti-tumor drug-related cardiotoxicity, myocardial protection

## Abstract

The development of anti-tumor drugs has notably enhanced the survival rates and quality of life for patients with malignant tumors. However, the side effects of these drugs, especially cardiotoxicity, significantly limit their clinical application. The cardiotoxicity associated with anti-tumor drugs has been a subject of extensive attention and research. Traditional to mitigate these side effects have included reducing drug dosages, shortening treatment duration, modifying administration methods, and opting for drugs with lower toxicity. However, either approach may potentially compromise the anti-tumor efficacy of the medications. Therefore, exploring other effective methods for anti-cardiotoxicity will be the focus of future research. The potential of traditional Chinese medicine (TCM) in managing cardiovascular diseases and cancer treatment has gained widespread recognition. TCM is valued for its minimal side effects, affordability, and accessibility, offering promising avenues in the prevention and treatment of cardiotoxicity caused by anti-tumor drugs. Among its constituents, flavonoids, which are present in many TCMs, are particularly notable. These monomeric compounds with distinct structural components have been shown to possess both cardiovascular protective properties and anti-tumor capabilities. In this discussion, we will delve into the classification of anti-tumor drugs and explore the underlying mechanisms of their associated cardiotoxicity. Additionally, we will examine flavonoids found in TCM and investigate their mechanisms of cardiovascular protection. This will include an analysis of how these natural compounds can mitigate the cardiac side effects of anti-tumor therapies while potentially enhancing overall patient health and treatment outcomes.

## 1. Introduction

Systematic research on anti-cancer drugs is generally believed to have begun in the 1940s. During this period, mechlorethamine was discovered at Yale University in the United States, and was shown to be effective in treating Hodgkin lymphoma and other forms of lymphoma and leukemia. Later, it was classified as an alkylating agent, which has been used to the present day. This is considered to be the beginning of the discovery and research of anti-tumor drugs [1]. Later researchers gradually discovered (or invented) various substances with anti-tumor activity from synthetic compounds, plants, animals, and microorganisms, thus developing sub disciplines such as cell kinetics, anti-tumor drug pharmacology, and tumor chemotherapy [2, 3, 4].

Malignant tumor and cardiovascular disease are serious threats to human life and health [5, 6]. Although the probability of cardiac tumor or metastatic tumor is small, myocardial damage from anti-tumor therapy is frequent, and has become the greatest risk factor to the prognosis and survival of cancer patients [7, 8]. Cardiotoxicity caused by anti-tumor drugs is mainly manifested in two aspects: myocardial cell dysfunction and cell death [5, 8, 9]. Its clinical manifestations include arrhythmia, myocardial ischemia, coronary artery disease, hypertension and myocardial dysfunction [9, 10]. Common antineoplastic drugs causing cardiotoxicity mainly include chemotherapeutic drugs (including anthracyclines, alkylating agent, anti-cellular microtubules, antimetabolics, platinum, etc.), and targeted drugs [11, 12, 13, 14, 15, 16]. Acute or subacute cardiotoxicity often arises during or within days to weeks of treatment, presenting as mild electrocardiogram (ECG) abnormalities, transient arrhythmias, and various conduction blocks, rarely leading to severe clinical symptoms [5, 8, 9]. Chronic cardiotoxicity is more prevalent and usually develops within a year of treatment. It is characterized by heart failure and/or cardiomyopathy, is often irreversible, and requires clinical treatment [11, 12, 13, 14, 15, 16]. Later stage cardiotoxicity occurs one year after the end of chemotherapy, mainly including concealed ventricular dysfunction, heart failure, and arrhythmia [11, 12, 13, 14, 15, 16]. This condition often manifests under increased cardiac stress, without evident symptoms in daily life [9, 10].

In the initial phase of cardiomyocyte toxicity induced by anti-tumor drugs, cardiomyocyte death primarily results from oxidative stress [17]. This process involves excessive production of reactive oxygen species (ROS), formation of metal ion complexes, inflammatory mediator release, cardiomyocyte homeostasis disruption due to mitochondrial abnormalities, DNA damage, and alterations to signaling pathways [17, 18, 19, 20, 21]. Ultimately, this process leads to cardiomyocyte death through mechanisms including necrosis, apoptosis, ferroptosis, autophagy, and pyroptosis [17, 18, 19, 20, 21]. Some anti-tumor drugs, such as cyclophosphamide, metabolize into toxic byproducts, such as acrolein, which can damage cardiomyocytes [12]. On the other hand, new immunosuppressants (programmed death 1 (PD-1)/programmed cell death-ligand 1 (PD-L1), etc.) disrupt cardiac immune homeostasis through immune pathways and cause heart damage [22].

Currently known cardioprotective drugs mainly include dexrazoxane, coenzyme Q10, angiotensin-converting enzyme inhibitors (ACEI) and β receptor blockers [23, 24, 25, 26]. Dexrazoxane is the only protective drug approved by Food and Drug Administration (FDA) for the prevention and treatment of cardiotoxicity of anti-tumor drugs, but it has not been widely used at present due to its high price [23]. Meanwhile, the clinical efficacy of ACEI and β receptor blockers remains relatively modest [23, 24, 25, 26]. All in all, the prevention and treatment of cardiotoxicity caused by anti-tumor drugs still needs further research.

The efficacy of traditional Chinese medicine (TCM) in the treatment of cardiovascular diseases and cancer has been widely recognized. TCM offers advantages including fewer side effects, affordability, and access, showing promising potential in the preventing and treating cardiotoxicity from anti-tumor drugs [18, 27, 28, 29, 30, 31, 32, 33, 34]. Various forms of TCM, including individual herbs, herbal compounds, patented Chinese medicines, and herbal extracts like Astragalus, Sini Decoction, Shengmai drink, Shengmai injection, Tongxinluo capsule, Yangxin granule, rutin, and curcumin have demonstrated significant preventive and therapeutic effects against cardiotoxicity induced by anti-tumor drugs [18, 27, 28, 29, 30, 31, 32, 33, 34]. This evidence underscores the substantial promise of TCM, suggesting it could be an effective approach to alleviate myocardial toxicity caused by anti-tumor drugs. However, identifying the active components in TCM remains an area that requires further research.

In this review, we provide a comprehensive summary of the onset, progression, clinical diagnosis and current treatment of cardiotoxicity induced by anti-tumor drugs, while discussing the preventive and therapeutic effects of flavonoids and their specific mechanisms. We emphasize the link between flavonoids and oxidative stress to regulate therapeutic targets and signaling pathways to improve the myocardial toxicity of anti-tumor drugs.

## 2. Cardiotoxicity of Anti-Tumor Drugs

**Fig. 1.**
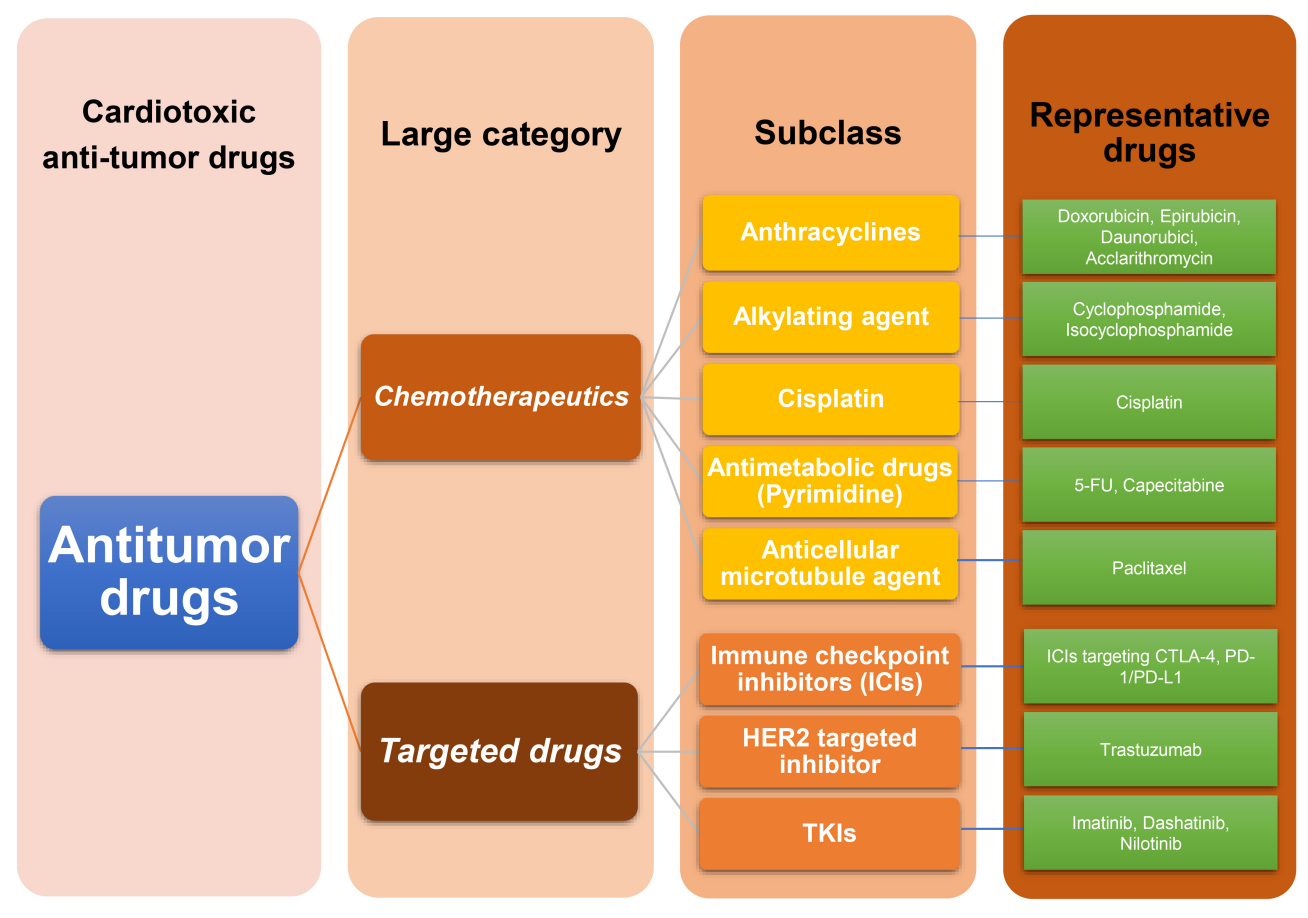
**Classification of anti-tumor drugs with cardiotoxicity**. Firstly, anti-tumor drugs with cardiac toxicity are mainly divided into two categories: chemotherapeutics and targeted drugs. Secondly, chemotherapy drugs can be divided into five subcategories: antibiotics, alkylating agents, cisplatin, antimicrobial drugs, and antibacterial microtubule agents. Targeted drugs are divided into three subcategories: ICIs, HER2 targeted inhibitors, and TKIs. Finally, each drug has its own representative drug, as detailed in the figure. ICIs, immune checkpoint inhibitors; HER2, human epidermal growth factor receptor-2; TKIs, tyrosine kinase inhibitors; 5-FU, 5-fluorouracil; CTLA-4, cytotoxic T-lymphocyte antigen 4; PD-1/PD-L1, Programmed Cell Death Protein 1/Programmed Death-Ligand 1.

Although nitrogen mustard was one of the earliest anti-tumor agents, there have been few cases of its associated cardiotoxicity. Current studies have found that anti-tumor drugs with cardiotoxicity mainly include anthracycline anti-tumor drugs, alkylating agent, platinum, cell microtubule drugs, anti-metabolic drugs, and targeted drugs [11]. Furthermore, cardiotoxicity caused by anti-tumor drugs has developed into the second largest cause of death for cancer patients [11, 35, 36, 37, 38, 39]. Representative anti-tumor drugs with corresponding cardiotoxicity are shown in Fig. 1.

### 2.1 Anthracyclines

Anthracyclines, derived from Streptomyces peucetius var caesius, are a group of chemotherapeutic drugs discovered in the 1970s [35, 36, 37]. They include doxorubicin, epirubicin, daunorubicin and aclacinomycin, which are widely used to treat hematological malignancies and solid tumors [35, 36, 37]. While they have a broad anti-tumor spectrum and are highly effective, they also cause significant side effects including hair loss, bone marrow suppression, and notably cardiotoxicity [11, 37]. Cardiotoxicity, the most severe and common side effect, shows dose-dependency and accumulates over time [11, 37]. The risk of heart failure increases with cumulative doses: 3% to 5% at 400 mg/$m^{2}$, 7% to 26% at 550 mg/$m^{2}$, and 18% to 48% at 700 mg/$m^{2}$ [9, 38]. In the initial stages of treatment, patients may experience mild arrhythmias, cardiac dysfunction, and cardiac hypertrophy [9, 11, 39]. With prolonged use, more severe effects like various malignant arrhythmias, a significant reduction in ejection fraction, and severe impairment of left ventricular function can occur [9, 11, 39].

Anthracyclines work by directly inhibiting topoisomerase II in cells, thus inhibiting DNA replication and transcription, and preventing ligase from repairing DNA [11]. During cellular metabolism anthracyclines interact with iron ions to form anthracycline-iron complexes, leading to a significant production of free radicals [40, 41, 42]. These radicals damage the structure and impair function of DNA, proteins, organelles, and cell membranes, culminating in cardiomyocyte death [40, 41, 42]. Additionally, the excessive production of ROS can lead to inflammation, calcium metabolism abnormalities, energy metabolism disorders, and mitochondrial damage. Importantly, these effects can exacerbate one another, creating a harmful cycle that ultimately results in irreversible cardiac damage [18, 43, 44].

### 2.2 Alkylating Agent

Cyclophosphamide and isocyclophosphamide are two common alkylating agents that are known to induce cardiotoxicity, with hemorrhagic necrotizing pericarditis being the most severe form of this toxicity [12, 45]. Cardiotoxicity occurs in 22% of patients at a cyclophosphamide dose of 170–180 mg/kg, and this incidence increases to 25% at a dose of 200 mg/kg, accompanied by higher mortality rates [12]. The underlying mechanism of this cardiotoxicity is primarily linked to endothelial cell damage, DNA base alkylation, and DNA damage caused by various toxic metabolites produced during liver metabolism [46, 47]. Among these metabolites, acrolein is the most cardiotoxic. It is known to damage cardiomyocytes, fibroblasts and endothelial cells, leading to autophagy, inflammation, oxidative stress and myocardial systolic dysfunction [12, 45].

### 2.3 Cisplatin

Cisplatin is highly active and effective against cancer, but its lack of specificity and significant side effects, including cardiovascular and renal toxicities, limit its use [13]. Similar to anthracyclines, cisplatin’s cardiotoxicity is dose- and concentration-dependent. Clinical manifestations of cisplatin cardiotoxicity include chest pain, palpitations, hypertension, chronic cardiac insufficiency, myocardial ischemia, ventricular hypertrophy, and myocardial infarction [48, 49]. A follow-up study found that 6% of patients treated with cisplatin developed acute myocardial infarction and angina pectoris, 7.1% developed coronary heart disease, and 33% had left ventricular diastolic dysfunction [50]. In addition, the study found that cisplatin significantly reduced coronary blood flow and heart rate, and increased left ventricular systolic blood pressure, and maximum left ventricular systolic rate [51]. The mechanism of its cardiotoxicity is also related to cytotoxicity, oxidative stress and inflammation [52, 53, 54].

### 2.4 Antimetabolic Drugs (Pyrimidine Antimetabolic Drugs)

The antimetabolic drugs 5-fluorouracil (5-FU) and capecitabine are pyrimidine analogs drugs known to induce cardiotoxicity. Vascular endothelial injury and coronary spasm are the key mechanisms of cardiotoxicity [14]. According to statistics, 2–10% of patients who receive 5-FU treatment will experience cardiovascular complications, and 2% of patients may have sudden cardiac death [55]. The mechanism of cardiotoxicity is currently thought to be related to inhibition of angiogenesis, endothelial dysfunction, abnormal energy metabolism, ROS and mitochondrial damage [56].

### 2.5 Anticellular Microtubule Agent

Paclitaxel is a natural anti-tumor drug, which can inhibit tumor growth by promoting tubulin polymerization and blocking cell mitosis. It is widely used in the treatment of digestive tract, ovarian, and cervical cancer [57, 58]. Studies have shown that paclitaxel can cause bradycardia, atrioventricular block, tachycardia, thrombus, myocardial ischemia and myocardial infarction [59, 60]. The mechanism may be related to the blocking of calcium and sodium ion channels in cardiomyocytes by taxine B [15]. The main causes of cardiotoxicity of paclitaxel are injury of endothelial cell function and promotion of thrombosis [61]. In addition, paclitaxel stimulates vaso-vagus nerve, increases parasympathetic sensitivity and causes hypothyroidism, which are the main reasons for bradycardia induced by paclitaxel [62].

### 2.6 Targeted Drugs

#### 2.6.1 Immune Checkpoint Inhibitors

Immune checkpoint inhibitors (ICIs) targeting cytotoxic T lymphocyte antigen 4 (CTLA-4) and PD-1/PD-L1 are crucial to tumor immunotherapy [63, 64]. Although cardiovascular toxicity associated with ICIs has been reported worldwide, it is often overlooked due to its low incidence, difficult diagnosis, and non-specific clinical manifestations [22]. Although the incidence of cardiotoxicity due to ICIs is only 0.27–1.14%, the fatality rate is high, and the myocarditis caused by ICIs combined treatment is more dangerous [22, 65, 66, 67]. Cardiotoxicity associated with ICIs is non-specific and includes myocarditis (the most common), pericarditis, arrhythmia, acute coronary syndrome, heart failure, vasculitis, cardiogenic shock, and cardiac arrest [65, 68, 69, 70, 71, 72, 73]. Unlike anthracyclines or platinum-based drugs, which are time and dose-dependent, ICIs may cause myocarditis after initial treatment, so it should be taken into account in clinical use [74].

After CTLA-4 and PD-1/PD-L1 are blocked, the proliferation and killing ability of immune cells are enhanced, which is more likely to cause tissue infiltration and trigger localized inflammation. This may be the root cause of myocardial immune damage induced by ICIs. Studies have shown that a large number of ${\mathrm{CD4}}^{+}$ and ${\mathrm{CD8}}^{+}$ T cell infiltrations were found in the target organs of patients, and the same high frequency T lymphocyte receptor sequences were also found in cardiac muscle and tumor tissue [65, 66, 75, 76, 77, 78, 79]. Additionally, in the heart muscle of PD-1-deficient mice, myosin autoantibody production was increased along with ${\mathrm{CD4}}^{+}$ and ${\mathrm{CD8}}^{+}$ T cell infiltration [80, 81]. These immune responses ultimately led to lymphocytic myocarditis, resulting in the eventual demise of the mice [80, 81]. CTLA-4 deficient mice have also been found to develop lymphoproliferative myocarditis with systemic inflammation and fatal multiple organ failure [82]. During a heart injury antigens are exposed to T lymphocytes, and the upregulation of immune checkpoints acts as an inhibitory signal for T cell invasion, but ICIs suppresses this cardioprotective measure [83]. Clones of the same T-cell receptor were found in the heart muscle, skeletal muscle, and tumor tissue of patients, suggesting that the body may have a T-cell response to a common antigen of tumor and muscle cell [67]. What’s more, ICIs combination therapy, such as CTLA-4 inhibitors combined with PD-1/PD-L1 inhibitors, resulted in an increased incidence of myocarditis with a mortality rate of up to 67% [66, 75]. Sex (male) and age (>65) are also important factors in the increased incidence and mortality of myocarditis [65, 84, 85].

#### 2.6.2 Other Targeted Drugs

Trastuzumab is the first human epidermal growth factor receptor-2 (HER2) targeted inhibitor to be used clinically [86]. Cardiotoxicity is an important adverse effect of trastuzumab, especially when combined with chemotherapy drugs [87]. The clinical manifestations are left ventricular systolic dysfunction characterized by decreased left ventricular ejection fraction and/or heart failure [88]. Studies showed that the mechanism of cardiotoxicity induced by trastuzumab is related to blocking HER2 signal, and ultimately affecting myocardial homeostasis, energy metabolism, and myofilament development [89].

Small molecule tyrosine kinase inhibitors (TKIs) are primarily used in the treatment of metastatic colorectal, breast, and kidney cancers [90, 91, 92]. The same mechanism that contributes to their anti-cancer efficacy also underlies their cardiotoxic effects. TKIs mainly inhibit vascular endothelial growth factor (VEGF), and reduce the activity of the vascular endothelial growth factor receptor (VEGFR) tyrosine kinase [90, 91]. This interference with vascular endothelial function leads to increasing peripheral vascular resistance and elevated blood pressure [90, 91]. Hypertension, the most common symptom, can also lead to arrhythmias, heart failure, myocardial ischemia, myocardial infarction and other adverse effects [90, 91]. Additionally, studies also demonstrated that small molecule TKIs caused the reduction of myocardial progenitor cells, which led to restrictive cardiomyopathy without significantly changing the myocardial structure, representing a potential new mechanism for its cardiotoxicity [92].

## 3. Clinical Diagnosis of Cardiotoxicity Caused by Anti-Tumor Drugs

In 2016, the European Society of Cardiology (ESC) categorized cardiovascular toxicity associated with tumor treatment into nine categories: myocardial dysfunction and heart failure, coronary heart disease, valvular disease, arrhythmia, hypertension, thromboembolic disease, peripheral vascular disease and stroke, pulmonary hypertension, and pericardial complications [93]. At present, the conventional clinical methods for diagnosing cardiotoxicity associated with anti-tumor drugs include electrocardiogram, echocardiography, biomarkers of myocardial injury, magnetic resonance and myocardial biopsy [73, 75, 94].

### 3.1 Electrocardiogram

The sensitivity and specificity of ECGs are not always sufficient, particularly in the early stages of cardiotoxicity, where significant changes may not be evident. In the intermediate stages of cardiotoxicity, various arrhythmias such as tachycardia, bradycardia, conduction block and atrial fibrillation may appear on the ECG [73, 75]. In the advanced stages of cardiotoxicity, especially in ICIs patients, complete block, ventricular tachycardia and ventricular fibrillation may occur [50]. However, it is important to recognize that normal ECG does not rule out the possibility of cardiotoxicity induced by anti-tumor drugs.

### 3.2 Echocardiography

Echocardiography is the preferred method for clinical evaluation of cardiac function due to its simplicity, convenience, affordability, and non-invasive features. Specific assessments can include ejection fraction, heart cavity size, and wall thickness. In most patients with anti-tumor cardiotoxicity, left ventricular function is impaired, as shown by reduced left ventricular ejection fractions (LVEF), abnormal lumen size and ventricular wall motion. However, in the early stage, echocardiography may be normal [73]. Assessing left ventricular diastolic function makes it easier to detect early heart damage [94].

In addition, speckle-tracking echocardiography (STE) can be used to visualize myocardial deformation during contraction, and calculate deformation rates, offering higher sensitivity to early and nuanced cardiac function changes [95, 96]. Tissue velocity doppler imaging (TVI)-derived parameters were shown to be independent of hemodynamic variables and to provide a more accurate and reproducible analysis of systolic and diastolic function, whose early changes suggest the development of cardiac insufficiency and increased mortality in patients with advanced disease [97, 98]. The overall long axial muscle strain is significantly reduced in patients with cardiotoxicity, which is more sensitive than LVEF, and is directly related to the decline in cardiac function after years of chemotherapy [73, 75, 99].

### 3.3 Myocardial Markers

In most patients with cardiotoxicity, serum levels of B-type natriuretic peptide (BNP) and N-terminal pro B-type natriuretic peptide (NT-proBNP) are elevated, with a sensitivity as high as 66–100% [100]. This increase may be attributed to chronic inflammation induced by cancer [100]. Cardiac troponin (cTn), a myocardial specific structural protein, is an early, sensitive, and specific marker for subtle myocardial damage; it offers reliable prediction of cardiotoxicity from anti-tumor drugs [101, 102]. The sensitivity of cTn to myocardial injury is remarkably high, at 94–100%, and levels of cTn ≥1.5 µg/L are indicative of a poor prognosis [103, 104, 105]. Since oxidative stress is an important mechanism of cardiotoxicity induced by anti-tumor drugs, myeloperoxidase (MPO) may become an independent risk factor associated with cardiotoxicity, although this hypothesis requires further clinical validation [106].

### 3.4 Magnetic Resonance

Cardiac magnetic resonance (CMR) is an important technique for evaluating left ventricular volume and function. It can also be used to evaluate myocardial strain, early microstructure and microvascular changes, and pericardial diseases. With high reproducibility, CMR can be used to comprehensively evaluate cardiac dysfunction related to cancer treatment, however the sensitivity is poor [73, 107]. Compared with echocardiography, CMR can also detect inflammation, edema, myocardial fibrosis, pericardial disease associated with heart dysfunction related to chemotherapy, and can be used as a complementary detection method [108].

### 3.5 Endomyocardial Biopsy

Endomyocardial biopsy remains the gold standard for the diagnosis of ICIs-associated myocarditis. However, due to is invasive nature, it is not the first choice in clinical settings. Pathologically, it is characterized mainly by focal or diffuse infiltration of ${\mathrm{CD8}}^{+}$ T cells, ${\mathrm{CD4}}^{+}$ T cells, and macrophages, along with the infiltration of other cell types, such as giant cells, neutrophil infiltration and eosinophils [67, 71]. These pathological features can occur in various parts of the heart, and a higher density is associated with increased lethality [109].

## 4. Current Treatment Plan

**Fig. 2.**
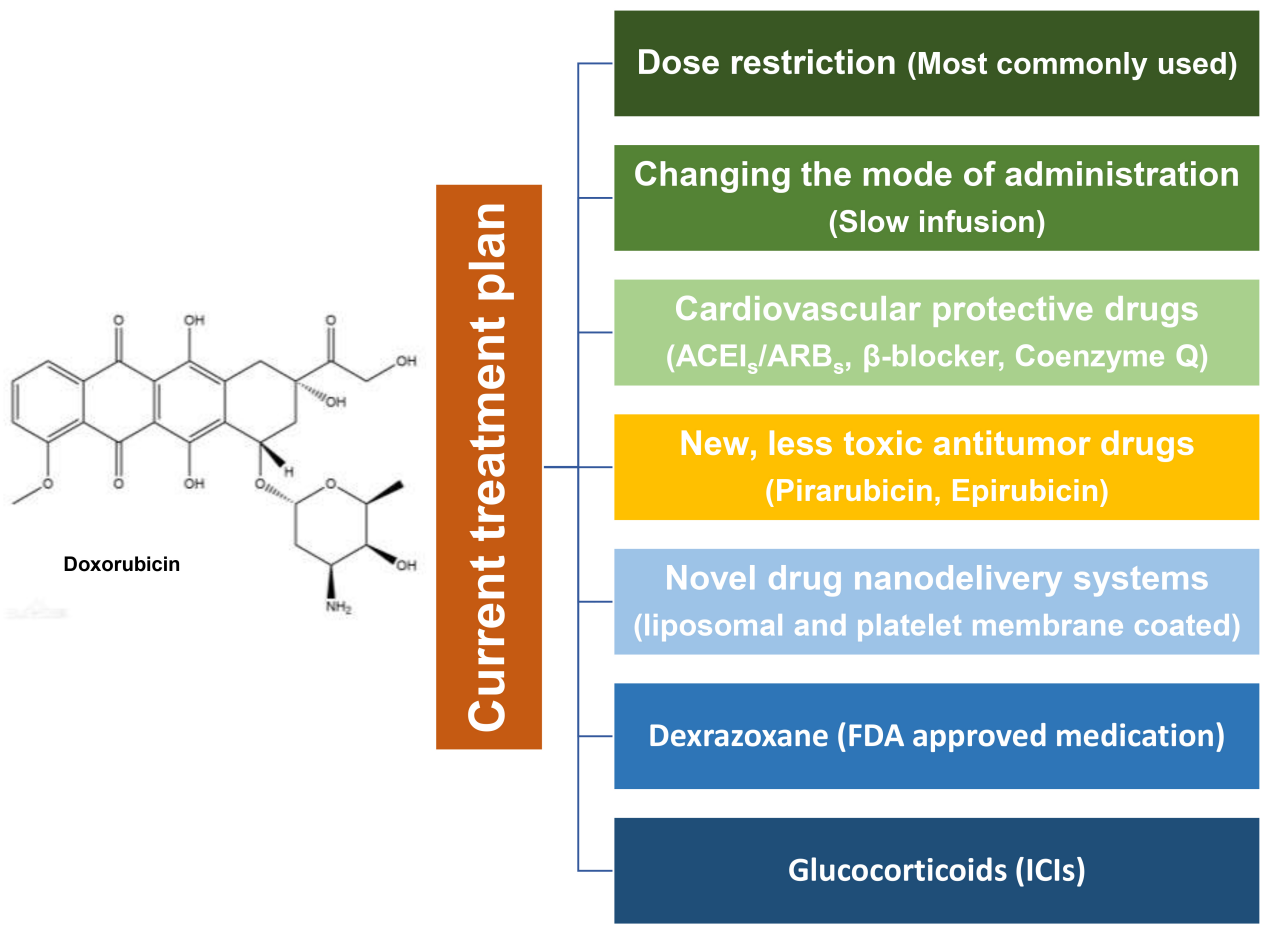
**The current treatment plan for cardiotoxicity caused by anti-tumor drugs**. The current treatment plans for anti-tumor drug cardiotoxicity are mainly divided into: dose restriction, changing the mode of administration, cardiovascular protective drugs, new, less toxic antibiotics or drugs, novel drug nanodelivery systems, dexrazoxane (FDA approved medicine), and Glucocorticoids (ICIs). ${\mathrm{ACEI}}_{s}$, angiotensin-converting enzyme inhibitor S; ${\mathrm{ARB}}_{s}$, angiotensin ii receptor blocker S; FDA, Food and Drug Administration; ICIs, immune checkpoint inhibitors.

The current treatment plan is shown in Fig. 2, which is detailed as follows.

Dose restriction is the most important measure for clinical prevention and treatment of anti-tumor drug-related cardiotoxicity. However, clinicians must remain aware that dose reduction will lead to a reduced therapeutic effect. Changing the mode of administration is another prevention method, but for dose-dependent and time-dependent drugs, at the same dose level, the ability to reduce side effects is limited [23, 24, 25, 26]. The use of new, less toxic anti-tumor drugs, such as pirarubicin, is also a way to prevent cardiotoxicity, but studies have found that pirarubicin can still cause serious cardiotoxicity. Dexrazoxan is currently the only specific drug approved for the treatment of chemotherapy-related cardiotoxicity [110]. However, its high price and potential inhibitory effect on the anti-tumor properties of other drugs limit its widespread use [110]. Other methods are mainly focused on symptom relief.

In cases of myocarditis caused by ICIs, which primarily induce immune-related myocarditis, the treatment approach differs from that for cardiotoxicity induced by cytotoxic drugs. Glucocorticoids are the primary and core treatment for ICIs-associated myocarditis. It is crucial to discontinue ICIs immediately upon the onset of early stage of ICIs-related myocarditis, and administer high-dose glucocorticoid therapy to patients (starting at 1~2 mg/kg, with potential escalation to 1 g/d and possibly in combination with other immunosuppressants). The dose should be gradually reduced following remission, until serum markers indicative of myocardial injury, such as BNP and cTn, normalize [111, 112, 113, 114]. Prophylactic use of glucocorticoids is not recommended because studies have shown that the use of glucocorticoids can diminish the anti-tumor efficacy of ICIs [111, 112, 113]. During treatment, glucocorticoid-induced adverse reactions such as blood glucose fluctuations, osteoporosis, deep vein thrombosis and infection should be noted [111, 112, 113].

In patients exhibiting mild symptoms of cardiotoxicity, re-administration of ICIs may be considered once biomarkers of myocardial damage return to normal levels. If ICIs-related cardiotoxicity reoccurs, switching to a different type of ICI should be considered [113, 115]. In the event of serious adverse reactions such as explosive myocarditis and severe arrhythmia, ICIs should be discontinued [116, 117]. In addition to glucocorticoids, other treatment methods include: (1) chemical agents, mainly mycophenolate and tacrolimus, used in combination with glucocorticoids [111, 113, 116]. (2) Biologics, mainly anti-thymocyte globulin, infliximab, alenzumab and abacipl [118, 119]. (3) Plasma exchange, which is generally used to remove cytokines and immune complexes in plasma during adverse reactions of the nervous system caused by ICIs [111, 113, 120, 121]. (4) Life support treatment, mainly including circulatory support, respiratory support and kidney replacement, mainly used for ICIs myocarditis critical type [117, 122].

## 5. The Potential of Traditional Chinese Medicine in Cardiotoxicity of Anti-tumor Drugs

Originating in China thousands of years ago, TCM has significantly influenced neighboring countries such as Japan, South Korea, North Korea, Vietnam. Its core theories, dating back about 2000 years, are centered around the concepts of Yin Yang and the five elements, TCM regards the human body as the unity of qi, shape and spirit [123, 124]. Through the method of “seeing, hearing and inquiring”, TCM explores the cause, nature and location of disease. They analyze the pathogenesis and changes to internal organs, meridians and joints, qi, blood and body fluids. The practice of TCM encompasses a range of therapeutic methods, including acupuncture, moxibustion, massage, and dietary therapy, all aimed at promoting healing and restoring balance to the human body [123, 124].

TCM has a longstanding history in the protection of heart function. Studies have shown that TCM can be used prophylactically to mitigate the cardiotoxic effects of anti-tumor drugs [123, 124]. Ginkgo biloba extract, at a dosage of 100 mg/kg/day, has been found effective in alleviated doxorubicin-induced cardiac injury [125, 126]. Astragalus ameliorates doxorubicin-induced cardiac function decline by up-regulating the sarcoplasmic/endoplasmic reticulum Ca2+ATPase 2a (SERCA2a) signaling pathway [127]. The use of Shengmai, as both a decoction and injection, has been observed preventing the cardiotoxicity of anti-tumor drugs by maintaining mitochondrial homeostasis [27, 28, 30]. Sini Decoction, a blend of aconite, ginger and licorice, has also been shown to improve heart failure caused by anti-tumor drugs [29]. Tongxinluo has been effective in preventing and treating dilated cardiomyopathy caused by anti-tumor drugs, working through the inhibition of ventricular remodeling, thus improving coronary microvascular function [31]. Yangxin Granules have been shown to reduce cardiac damage induced by anti-tumor drugs through the inhibition of oxidative stress and apoptosis mediated by the protein kinase B (AKT), glycogen synthase kinase 3 β (GSK3β), and β-catenin signaling pathway [32].

These findings highlight the significant potential of TCM in the prevention and treatment of cardiotoxicity associated with anti-tumor drugs. However, the components of TCM are complex, typically comprising natural extracts with numerous active ingredients, poses a challenge. Identifying specific TCM monomers with cardioprotective effects among these active ingredients is crucial for advancing this field.

## 6. The Potential of Flavonoids in Cardiotoxicity of Anti-Tumor Drugs

### 6.1 Flavonoids in Traditional Chinese Medicine and Cardiovascular Diseases

**Fig. 3.**
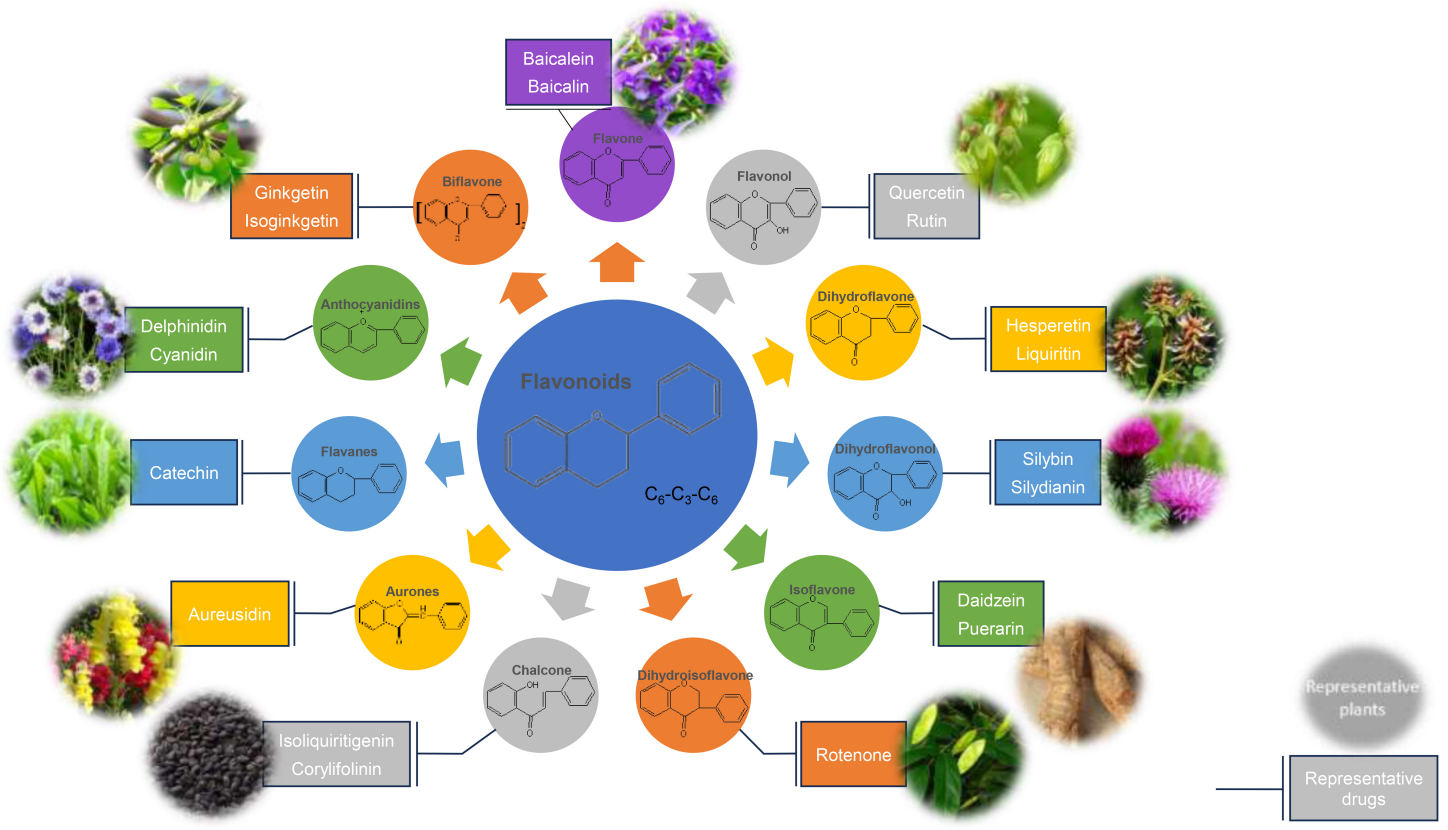
**The main structural basis and classification of flavonoids, representative drugs, and main plant sources**. The main structural basis of flavonoids is $C_{6}$-$C_{3}$-$C_{6}$, which can be mainly divided into 11 types of flavonoid structures, each with its representative drugs and main plant sources.

Flavonoids are compounds characterized by a $C_{6}$-$C_{3}$-$C_{6}$ structure, consisting of two benzene rings linked by a three carbon atom chain [128]. They are widely found in plants as natural secondary metabolites [128]. Based on the oxidation level of the $C_{3}$ structure and the linkage position of the B-ring, flavonoids can be categorized into several groups: flavonoids and flavonols, flavanones and flavanones, isoflavones, isoflavones, chalcones, dihydrochalcones, orange ketones, flavanones and flavanols [18, 33, 34]. The classification of flavonoids can also be seen in Fig. 3 or Table 1. Many monomeric compounds in TCM that are metabolically active in the human body include flavonoids, such as baicalein, schisandrin B, rutin, quercetin, puerarin, hyperin, total flavonoids of ginkgo biloba leaves, hyperin, silymarin, luteolin, total flavonoids of hippophae rhamnoides, daidzein, and kaempferol [18, 33, 34]. These compounds are known for their beneficial effects in the prevention and treatment of cardiovascular diseases, such as arteriosclerosis, hyperlipidemia, hypertension, and myocardial infarction [18, 33, 34].

**Table 1.** **The main structural basis and classification of flavonoids, representative drugs, and main plant sources**.

Structural basis	Classification	Representative drugs	Main plant sources	Content
$C_{6}$-$C_{3}$-$C_{6}$	Flavone	Baicalein	Scutellaria baicalensis Georgi	8% (root)
		Baicalin		
	Flavonol	Quercetin	Rutagraveolens L.	6% (flower)
		Rutin		
	Dihydroflavone	Hesperetin	Citrus reticulata Blanco	Unknown.
		Liquiritin	Glycyrrhiza uralensis Fisch.	0.5∼2%
	Dihydroflavonol	Silybin	Silybum marianum L. Gaertn.	0.60% (fruit)
		Silydianin		
	Isoflavone	Daidzein	Pueraria lobata(Willd) Ohwi	2.4% (root)
		Puerarin		
	Dihydroisoflavone	Rotenone	Derris trifoliata Lour.	12% (root)
	Chalcone	Isoliquiritigenin	Glycyrrhiza uralensis Fisch.	0.2∼0.5%
		Corylifolinin	Psoralea corylifolia L.	0.7% (fruit)
	Aurones	Aureusidin	Antirrhinum majus L.	Unknown.
	Flavanes	Catechin	Acacia catechu (L. f.) Willd.	10% (branch)
	Anthocyanidins	Delphinidin	Consolida ajacis (L.) Schur	1% (seed)
		Cyanidin	Centaurea cyanus L.	Unknown.
	Biflavone	Ginkgetin	Ginkgo biloba L.	0.4% (leaf)
		Isoginkgetin		

The main structural basis of flavonoids is $C_{6}$-$C_{3}$-$C_{6}$, which can be mainly divided into 11 types of flavonoid structures, each with its representative drugs and main plant sources.

### 6.2 Flavonoids in Traditional Chinese Medicine and Oxidative Stress

Oxidative stress is a harmful state that occurs when there is an accumulation of ROS, either within the body (*in vivo*) or in an experimental setting (*in vitro*) [129]. This accumulation can be due to physiological stressors or pathological conditions [129]. While ROS at physiological concentrations play a crucial role in defending against external infectious agents, the excessive accumulation of ROS can cause damage to cardiovascular endothelial cells and stromal cells [129]. This damage can induce apoptosis, promote inflammation, and lead to a variety of cardiovascular and cerebrovascular diseases [129].

Natural flavonoids often contain hydroxyl, methoxy, alkoxy, and isopentenyl groups on their parent nucleus. The phenolic hydroxyl groups in flavonoids can react with peroxyl free radicals to form flavonoid free radicals, which then interact with other free radicals [128]. This process effectively terminates the free radical chain reaction and inhibits oxidative stress [130]. Studies have shown that flavonoids have the ability to resist oxidative stress and scavenge free radicals [128, 130]. The body’s intrinsic antioxidant enzyme system primarily consists of superoxide dismutase (SOD), catalase (CAT), and glutathione peroxidase (GSH-Px) [128, 129, 130, 131]. Studies have found that flavonoids can enhance the activity of these antioxidant enzymes, thereby aiding in the clearance of ROS, helping to prevent and mitigate cardiovascular diseases [131]. Furthermore, flavonoids are also known to exert their anti-oxidative stress effects by modulating the nuclear factor erythroid2-related factor 2 (Nrf2)/heme oxygenase-1 (HO-1) signaling pathway [132, 133, 134].

### 6.3 Flavonoids in Traditional Chinese Medicine and Cardiotoxicity of Anti-Tumor Drugs

Studies have shown that oxidative stress plays an important role in cardiotoxicity induced by anti-tumor drugs [17]. Rutin is a natural flavonoid compound, which is widely found in various plants, and has anti-oxidant properties, promoting the protection of cardiomyocytes [135, 136]. Rutin is known to regulate intracellular ROS levels and inhibit myocardial oxidative damage by regulating the micro-RNA (miR-125b-1-3p) mediated JunD proto-oncogene (JunD) signaling pathway [137]. In addition, rutin inhibited ROS production and apoptosis by regulating interactions between the transforming growth factor-β1 (TGF-β1) and p38 mitogen-activated protein kinase (p38 MAPK) signaling pathways, thereby ameliorating cardiotoxicity induced by pirarubicin [138]. Studies have shown that astragaloside IV alleviates cardiomyocyte apoptosis, myocardial fibrosis and cardiac dysfunction caused by adriamycin [139]. The protective mechanism involves the inhibition of oxidative stress mediated by nicotinamide adenine dinucleotide phosphate oxidases 2 and nicotinamide adenine dinucleotide phosphate oxidases 4 (NOX2 and NOX4) [139]. Luteolin has been found to ameliorate doxorubicin-induced cardiotoxicity through mechanisms that regulate and calcium overload [140]. Quercetin can counteract cardiotoxicity induced by anti-tumor drugs by regulating ROS production, mitochondrial permeability conversion pore opening, and apoptosis [141]. Studies have also shown that the combination of quercetin and sitagliptin (10 mg/kg) enhances the protective effect against anthracycline-induced cardiotoxicity [142]. Studies showed that hesperidin improves doxorubicin-induced cardiotoxicity by regulating oxidative stress and apoptosis [143, 144].

In addition, our previous study established that schisandrin B, a flavonoid compound, effectively mitigates the cardiotoxicity of anti-tumor drugs, particularly pirarubicin [18]. Schisandrin B is the most abundant flavonoid monomer in schisandrin and also plays a pivotal role. Studies have shown that schisandra B improves a series of cardiac dysfunction manifestations (abnormal echocardiography and electrocardiogram, abnormal biochemical markers of myocardial damage, abnormal increase of ROS in cardiac tissue, and cardiomyocyte apoptosis) in rats experiencing anthracycline-induced cardiotoxicity [18]. The primary mechanism behind these effects appears to be schisandrin B’s ability to improve mitochondrial homeostasis, inhibit excessive cytochrome C production, and suppress the apoptosis pathway [18]. These findings underscore the potential of flavonoids in the preventing and treating myocardial toxicity induced by anti-tumor drugs.

## 7. Perspectives

The treatment of tumors remains a significant challenge in modern medicine. Although new anti-tumor drugs continue to emerge, studies have found that both anthracyclines and immunosuppressants have serious cardiotoxic effects, and there is a lack of specific drugs to counter these effects. It is worth noting that the potential of TCM in the treatment of cardiovascular diseases and anti-tumor has been widely recognized, and it has the advantages of fewer side effects, affordability, and accessibility, presenting a promising approach in the prevention and treatment of cardiotoxicity caused by anti-tumor drugs [5, 6, 7, 8, 9, 10, 11, 12, 13, 14, 15, 16, 17, 18, 19, 20, 21, 22, 27, 28, 29, 30, 31, 32, 33, 34]. Flavonoids, commonly found in many TCMs, are monomers with clear structural components, and have demonstrated both cardiovascular benefits and anti-tumor properties [27, 28, 29, 30, 31, 32, 33, 34, 123, 124, 125, 126, 127, 128, 129, 130, 131, 132, 133, 134, 135, 136, 137, 138, 139, 140, 141, 142, 143, 144]. In addition, there have been no reports on the side effects of naturally occurring flavonoids, making them a particularly exciting prospect. However, further studies are required to fully explore the clinical advantages and full potential of flavonoids.

Looking to the future, it is hoped that more effective TCM-based strategies for preventing and treating cardiotoxicity induced by anti-tumor drugs will emerge. Ideally, these TCM approaches will be integrated with Western medicine to provide substantial clinical benefits to a broad spectrum of cancer patients.

## 8. Conclusions

In summary, flavonoids are the main active ingredients in various TCMs, demonstrate extensive therapeutic effects in cardiovascular diseases, especially those related to chemotherapy-induced cardiotoxicity. This article summarizes and narrates aspects of TCM, with emphasis on the existing research of flavonoids. The aim is to offer insights for future basic and clinical research, and to establish a systematic theoretical foundation for the clinical application of flavonoids and related TCM. This synthesis of knowledge and research could potentially guide more effective and integrative treatment approaches in the realm of cardiology, especially for patients undergoing cancer therapy.
